# Incidence of fractures among patients receiving medications for type 2 diabetes or chronic obstructive pulmonary disease and glucocorticoid users according to the National Claims Database in Japan

**DOI:** 10.1007/s11657-021-00968-9

**Published:** 2021-06-30

**Authors:** Saeko Fujiwara, Shigeyuki Ishii, Takahiko Hamasaki, Nobukazu Okimoto

**Affiliations:** 1 Department of Pharmacy, Yasuda Women’s University, 6-13-1 Yasuhigashi, Asaminami-ku, Hiroshima, 731-0152 Japan; 2 Tokyo University of Pharmacy and Life Sciences, Tokyo, Japan; 3 Chugoku Rosai Hospital, Hiroshima, Japan; 4 Okimoto Clinic, Hiroshima, Japan

**Keywords:** Hip fracture, Clinical vertebral fracture, Type 2 diabetes, Glucocorticoids, Chronic obstructive pulmonary disease, Radial fracture

## Abstract

***Summary*:**

According to information from the National Health Insurance and Claims database, the risk for hip, radius, and clinical vertebral fractures was higher among patients receiving medication for type 2 diabetes, COPD, or glucocorticoids than among the whole Japanese population after middle age.

**Purpose:**

The aim of this study was to determine the incidence of fractures among patients receiving medications for type 2 diabetes or chronic obstructive pulmonary disease (COPD) and using glucocorticoids (GC) according to the National Database of Health Insurance Claims (NDB) in Japan.

**Methods:**

We obtained data on the number of fractures and patients receiving medications for type 2 diabetes, COPD, or GC from the NDB. The claims data included sex, age group, injury/illness name, hospitalization, outpatient, surgery/medical treatment, and drugs used between January and December 2017.

**Results:**

The risk of hip fracture was higher among patients receiving medications for diabetes or COPD and GC users than in the Japanese population, with standardized incidence ratios (SIRs) of 1.71 (95% confidence interval [CI]1.6–1.75), 1.35 (95% CI 1.28–1.42), and 1.62 (95% CI 1.53–1.71) in men and 1.81 (95% CI 1.79–1.84), 1.67 (95% CI 1.54–1.80), and 1.71 (95% CI 1.66–1.76) in women, respectively. There was also a significantly higher incidence of radial fractures in women and clinical vertebral fractures in both men and women. A greater risk of hip fracture was found among diabetic patients starting in their late 40 s.

**Conclusions:**

Real-world data revealed that the incidence of hip, radius, and clinical vertebral fractures was significantly higher among patients receiving medications for diabetes or COPD and GC users than in the Japanese population after middle age.

**Supplementary Information:**

The online version contains supplementary material available at 10.1007/s11657-021-00968-9.

## Introduction

Fractures associated with osteoporosis reduce physical activity and the quality of life among the elderly and are one of the major reasons for the requirement of long-term care. Although the age-adjusted incidence rates for hip fracture in both sexes in Japan did not change in the past quarter-century, the number of patients with new hip fractures increased 2.5 times from 1992 to 2017 [1]. Japan is the first country to host a super-elderly society; the number of elderly people aged ≥ 65 years accounted for 28.5% of the total population in 2020, and this percentage is projected to exceed 30% of the population by 2025 [2]. Thus, the number of elderly patients with type 2 diabetes, chronic obstructive pulmonary disease (COPD), and taking glucocorticoids (GC) is also projected to increase. Furthermore, type 2 diabetes is a typical lifestyle-related disease, the incidence of which is rapidly increasing because of the westernization of dietary habits and lifestyle in Japan. Recently, epidemiological and clinical studies have indicated that diabetes [3–5] and COPD [6, 7] are risk factors for osteoporotic fractures. In addition, GCs are known to cause osteoporosis and increase the risk of fracture [8]. These diseases are being treated by internists, however, most of whom rarely consider the medical history of fractures and pay little attention to the risk of fractures. To reduce osteoporotic fracture, it is important to recognize the risk of fractures among these diseases in the real world. However, there have been no reports on the incidence of fractures using real-world data among patients receiving medications for type 2 diabetes or COPD and GC users in Japan. The medical insurance system in Japan is a “universal insurance system” in which individuals are obliged to take out some kind of public medical insurance. Information on this system was stored as the National Database of Health Insurance Claims and Specific Health Checkups of Japan (NDB), which is managed by the Ministry of Health, Welfare, and Labor (MHLW). Furthermore, the NDB includes information from 98% of individuals receiving insurance-covered healthcare services in Japan [9].

This study aimed to determine fracture incidence among patients receiving medications for type 2 diabetes or COPD and GC users according to NDB data and to compare the incidence with that in the whole Japanese population.

## Methods

Aggregated claims data, including sex, age group, injury/illness name (ICD), hospitalization, outpatient, surgery/medical treatment, and drugs used between January and December 2017 were obtained from the NDB for this study. To clarify the periods of steroid use, claims data from 2016 were reviewed in addition to data from 2017. To improve the accuracy of diagnosis of disease and fracture, disease codes with billing codes of treatments or medical intervention codes, such as treatment/operation/hospitalization, were extracted. Multiple cases for the same person were counted as one case using the patient’s name.

### Ascertainment of patients receiving medications for type 2 diabetes or COPD and GC users

We defined type 2 diabetes as having diagnostic codes for type 2 diabetes (ICD 10: E11, E14, and R730) and a prescription of oral and injectable drug codes recognized as standard diabetes drug treatment. COPD was defined as having an ICD code of COPD (J43, J44) and a prescription of a long-acting muscarinic antagonist (LAMA) and/or long-acting β agonist (LABA). Steroid users were defined as having a prescription for oral GC for 3 months or more using data from 2016 and 2017.

### Ascertainment of fracture

We defined patients with hip fracture events as patients with both hip fracture and hip operation codes in the same year (Supplementary 1). The data were from a single year without a washout period, as there was an exact date on the extracted data when the patients were first diagnosed. Radial fractures were considered having both ICD codes (Supplementary 2) and medical procedure codes for invasive or non-invasive fixation of the forearm or surgery. A clinical vertebral fracture incident was defined as having ICD codes for vertebral fracture and the procedure code for a cast or trunk orthosis (Supplementary 3).

### Population data in Japan

Data on the Japanese population by sex and 5-year age group in 2017 were based on the published vital statistics survey of the Ministry of Health, Labor and Welfare (https://www.mhlw.go.jp/toukei/saikin/hw/jinkou/kakutei17/index.html.) [10].

### Statistics analyses

We calculated the sex- and age-group-specific incidence rates of fractures using both census data for the Japanese population and the NDB. To test the significance of the difference in sex-specific incidence rates between each disease (type 2 diabetes, COPD, and GC users) and the Japanese population, we calculated the *Z* score as follows:

$$Z=A/\surd B.$$

*A* is the difference in incidence rate between the Japanese population and disease by age group. *B* is the variance of difference in incidence rate *(B*) between the Japanese population and disease by age group.

If *Z* >  + 1.95 or <  − 1.95, the difference in incidence rates was considered statistically significant.

The standardized incidence ratio (SIR) among patients receiving medications for type 2 diabetes or COPD and GC users was calculated as follows:

$$\mathrm{S}\mathrm{I}\mathrm{R}=\frac{D}{\sum \left(I\times P\right)}$$

*D* is the observed number of fractures among patients receiving medications for type 2 diabetes or COPD and GC users. *I* is the sex and age group-specific incidence rates of fracture in the Japanese population. *P* is the sex and age group-specific population.

The standardized incidence ratios (SIRs) for diabetic patients and steroid users aged >= 40 years were estimated. The SIR for COPD patients was estimated among men and women aged >= 60 years because of the limited number of patients aged < 60 years and fracture among these patients.

Confidence interval of the SIR was calculated as follows:

$$\mathrm{L}\mathrm{o}\mathrm{w}\mathrm{e}\mathrm{r}=\mathrm{S}\mathrm{I}\mathrm{R}{(1-\frac{1}{9D}-\frac{1.96}{3\sqrt{D}})}^{3}$$

$$\mathrm{U}\mathrm{p}\mathrm{p}\mathrm{e}\mathrm{r}=\mathrm{S}\mathrm{I}\mathrm{R}\left(D+1\right){(1-\frac{1}{9\left(D+1\right)}+\frac{1.96}{3\sqrt{D+1}})}^{3}$$

*D* is the observed number of fractures among patients with type 2 diabetes, COPD, and steroid users.

## Ethical considerations

This study was approved by the Ethics Committee of Yasuda Women’s University. We included only cross-tabulation data provided by the Ministry of Health, Labor, and Welfare Insurance Bureau. These data were completely anonymous; thus, informed consent was not required.

## Results

### Percentages of patients receiving medications for type 2 diabetes or COPD and GC users (Table 1)

**Table 1:** Percentages of type 2 diabetes or COPD on medication, and glucocoiticoide users among Japanese population

Disease	Sex	Age (years)											
		40–44	45–49	50–54	55–59	60–64	65–69	70–74	75–79	80–84	85–89	90 +	60 +
Type 2 diabetes (%)	Men	2.3	3.9	5.8	8.1	10.9	13.5	14.6	15.9	15.7	13.1	9.1	13.6
	Women	0.9	1.5	2.4	3.8	5.7	7.8	9.1	10.3	10.5	9.4	7.1	8.5
COPD (%)	Men					0.6	1.2	2	2.8	3.4	3.7	2.9	1.9
	Women					0.1	0.2	0.3	0.4	0.5	0.4	0.3	0.3
Glucosorticoide users (%)	Men	0.4	0.5	0.6	0.8	1	1.3	1.7	2.2	2.5	2.6	2.4	1.7
	Women	0.8	0.9	1.1	1.2	1.4	1.5	1.7	2.1	2.3	2.3	2	1.8

Among the Japanese population aged ≥ 60 years old, 13.6% of the men and 8.5% of the women had type 2 diabetes, and that percentage increased with age, with the highest being 15.9% in men aged 75–79 years and 10.5% in women aged 80–84 years. Furthermore, 1.9% of men and 0.3% of women were receiving medications for COPD; however, those percentages increased to 3.7% in men aged 86–89 years and 0.5% in women aged 80–85 years, which was the highest percentage.

Regarding the use of GS, 1.7% of men and 1.8% of women aged ≥ 60 years, and 2.6% of men and 2.3% of women aged in their late 80 s were receiving GCs for at least 3 months.

### Incidence of fractures among patients with type 2 diabetes or COPD on medication, and GC users

#### Type 2 diabetes on medication

There were 169,200 hip fractures, 88,700 radius fractures, and 85,500 clinical vertebral fractures in the Japanese population in 2017. Among these, 17.4%, 10.7%, and 13.3% of hip, radius, and clinical vertebral fractures, respectively, developed among patients with type 2 diabetes (Tables 2, 3, and 4).

**Table 2:** Hip fracture incidence (/100,000 person-year)among type 2 diabetes or COPD on medication, and glucocorticoid users

Sex	Age	All population	Diabetes	COPD	Glucocorticoid
Incidence(/100,000 person-year)					
Men	40–44	4.8	10.3		9.7
	45–49	7.3	22*		17.2
	50–54	12.1	32.7*		33.5
	55–59	18.1	45.1*		73.5*
	60–64	30.4	67.5*	30.9	87.7*
	65–69	52.2	117.1*	103.3*	146.9*
	70–74	88.1	192.2*	163.6*	199.6*
	75–79	176.2	304.6*	307*	342.4*
	80–84	377.7	615.3*	492.8*	637.7*
	85–89	740.3	1081.8*	919.8*	929.3*
	90 +	1213.7	1716*	1231	1327.3
Women	40–44	3.3	7.2		16.4
	45–49	6	22.3*		23.4*
	50–54	17.6	52.8*		67.2*
	55–59	32.4	86.2*		116.0*
	60–64	54.2	145.6*	96.2	238.9*
	65–69	100.7	252.5*	242.2*	342.6*
	70–74	174.1	417.6*	351.2*	535.5*
	75–79	379.3	808.0*	787.8*	913.0*
	80–84	885.7	1601.1*	1532.4*	1587.3*
	85–89	1634.2	2647.0*	2215.4*	2274.7*
	90 +	2430.8	3827.7*	2693.5	2821.7*
Total number of fracture(% among all hip fractures)		169,200	29,500(17.4%)	2200(1.3%)	6100(3.6%)

^*^Significant high incidence compared with whole population

**Table 3:** Radius fracture incidence (/100,000 person-years)among type 2 diabetes, COPD on medication, and glucocorticoid users

Sex	Age	All population	Diabetes	COPD	Glucocorticoid
Incidence(/100,000 person-year)					
Men	40–44	20.6	31.7		14.6
	45–49	24.6	31.3		25.7
	50–54	28.1	40.4*		62.8*
	55–59	29.7	39.8*		52.5
	60–64	32.5	42.9*	17.6	49*
	65–69	37.8	48.3*	46.3	39.5
	70–74	44.2	59.1*	59.1	57.7
	75–79	55.6	76.7*	42	75.2*
	80–84	70.5	94.8*	68.8	68.6
	85–89	93.8	116.9*	131.1*	106.5
	90 +	95.4	95.2	103.7	91.2
Women	40–44	13.8	19.1		24.6
	45–49	20.2	41.6*		35.1
	50–54	53.7	68.3		74.1
	55–59	120.9	127.2		155.4
	60–64	173.9	202.4*	173.1	222.2*
	65–69	216.7	262.6*	287	250.1
	70–74	267.4	344.6*	270.7	304*
	75–79	338	409*	441.2*	348.2
	80–84	381.8	440.7*	479.7*	400.3
	85–89	405.6	482*	625.6*	376.5
	90 +	280.3	333.2*	424.2*	225.4#
Total number of fracture		88,700	9500(10.7%)	500(0.6%)	1700(1.9%)

^*^Significant high incidence compared with whole population

^#^Significant loe incidence

**Table 4:** Spine fracture incidence (/100,000 person-years)among type 2 diabetes or COPD on medication, and glucocorticoid users

Sex	Age	All population	Diabetes	COPD	Glucocorticoid
Men	40–44	4.9	12.1*		19.4*
	45–49	6.1	13.2*		42.9*
	50–54	9.5	15.3*		50.3*
	55–59	13.9	24.7*		80.5*
	60–64	24.8	37.4*	83.8*	149.7*
	65–69	43.7	60.2*	114*	230.6*
	70–74	76.2	111.5*	171.8*	313.4*
	75–79	147	183.1*	253*	535.8*
	80–84	253	291.0*	362.1*	685.9*
	85–89	359.8	397.0*	480.6*	764.7*
	90 +	413.3	473.8*	587.8*	705.1*
Women	40–44	3	4.8*		19.1*
	45–49	4.6	14.9*		32.8*
	50–54	11.7	30*		92.6*
	55–59	25.7	50.7*		124.8*
	60–64	53.8	97.7*	134.6	244.5*
	65–69	103.5	165.9*	260.1*	440.3*
	70–74	183.6	265.8*	395.1*	616.5*
	75–79	332.3	424.9*	573.5*	931.1*
	80–84	475.3	556.3*	746.2*	1089.7*
	85–89	531.8	651.3*	676.9*	1026.1*
	90 +	420.1	541.1*	572.6*	720.5*
Total number of fracture		85,500	11,400(13.3%)	4700(5.5%)	6100(7.1%)

^*^Significant high incidence compared with whole population

The age-specific incidence of fractures of type 2 diabetes patients and the Japanese population was compared. The incidence of hip and radius fractures, respectively, was significantly higher in both men and women receiving medication for diabetes (Tables 2 and 3). Furthermore, men and women aged ≥ 40 years who were treated for diabetes had a significantly higher incidence of spinal fractures (Table 4).

#### COPD patients on medication

The incidence of hip and clinical vertebral fractures was significantly higher in both men and women aged ≥ 60 years who were undergoing COPD drug treatment (Tables 2 and 4). Radius fracture incidence was significantly higher in women aged ≥ 75 years, but there was no statistical difference in the incidence among men of different age groups, except in the 85–89-year age group (Table 3).

### GC users

Compared to the incidence in the Japanese population, the incidence of hip fractures was significantly higher in women starting in their late 40 s and in men starting in their late 50 s (Table 2). The incidence of distal radius fracture was significantly greater in some age groups of both men and women but not in all age groups (Table 3). The clinical vertebral fracture incidence was significantly higher in both male and female GC users aged ≥ 40 years (Table 4).

### Standardized incidence ratios

We estimated the SIRs in patients receiving medications for type 2 diabetes. Among those aged ≥ 40 years, the SIRs of hip, radius, and clinical vertebral fractures were 1.71 (95% confidence interval [CI] 1.6–1.75), 1.32 (95% CI 1.26–1.38), and 1.23 (95% CI 1.21–1.29) in men and 1.81 (95% CI 1.79–1.84), 1.20 (95% CI 1.18–1.23), and 1.30 (95% CI 1.27–1.33) in women, respectively (Table 5).

**Table 5:** Standardized incidence ratio of fracture incidence

Disease	Sex	Standardized incidence ratio of fracture incidence (95% Confidence Interval)								
		Hip			Radius			Spine		
Type 2 diabetes	Men	1.71	(1.60,	1.75)	1.32	(1.26,	1.38)	1.25	(1.21,	1.29)
	Women	1.81	(1.79,	1.84)	1.20	(1.18,	1.23)	1.30	(1.27,	1.33)
COPD	Men	1.35	(1.28,	1.42)	1.08	(0.95,	1.23)	1.60	(1.51,	1.71)
	Women	1.67	(1.54,	1.80)	1.28	(1.13,	1.43)	1.67	(1.50,	1.85)
GC	Men	1.62	(1.53,	1.71)	1.22	(1.07,	1.38)	3.06	(2.90,	3.22)
	Women	1.71	(1.66,	1.76)	1.07	(1.02,	1.13)	2.60	(2.51,	2.69)

Standardized incidence ratio (SIR) was estimated among patients receiving medication for diabetes and GC users aged 40 yrs and over, and patients receiving medication for COPD aged 60 yrs and over

Among those aged ≥ 60 years on medication for COPD, the SIRs of hip, radius, and clinical vertebral fractures were 1.35 (95% CI 1.28–1.42), 1.08 (95% CI 0.95–1.75), and 1.60 (95% CI 1.51–1.71) in men and 1.67 (95% CI 1.54–1.80), 1.28 (95% CI 1.13–1.43), and 1.67 (95% CI 1.50–1.85) in women, respectively (Table 5).

The SIRs of hip, radius, and clinical vertebral fractures were 1.62 (95% CI 1.53–1.71), 1.22 (95% CI 1.07–1.38–), and 3.06 (95% CI– 23.90–3.22) among men using GCs, whereas they were 1.71 (95% CI 1.66–1.76), 1.07 (95% CI 1.02–1.13), and 2.60 (95% CI 2.51–2.69) among women using GCs, respectively (Table 5).

Except for radius fracture among men with COPD, the risks of the three fractures were statistically higher among patients receiving medications for type 2 diabetes or COPD and GC users than among the Japanese population aged ≥ 40 years.

## Discussion

To the best of our knowledge, this is the first comprehensive study on the incidence of hip, distal radius, and clinical spine fractures among patients receiving medications for type 2 diabetes or COPD and GC users according to National Health Insurance claims data in Japan. This study revealed a higher real-world incidence of fractures in patients aged ≥ 40 years who were receiving medications for type 2 diabetes, COPD, or GCs for more than 3 months.

In Japan, with an aging population, the number of patients with new hip fractures increased by 2.5-fold in the past quarter-century [1]. A range of pharmaceutical interventions has been shown to be effective in reducing fracture risk in postmenopausal women with osteoporosis. However, patients diagnosed with osteoporosis are often not treated. In Japan, 4.8% of men aged 70–79 years and 23.7% of women aged 70–79 years are treated for osteoporosis [11]. This study showed that 17.4% of hip fractures developed among patients with type 2 diabetes. If all diabetic patients with high risk of fracture receive medications for osteoporosis, an ~ 8% reduction of cases of hip fracture would be anticipated annually in Japan. Furthermore, COPD are prevalent among men, but the treatment rates of osteoporosis are very low among men. Therefore, we believe that physicians who routinely see patients with type 2 diabetes or COPD should proactively identify patients with osteoporosis or high risks of fracture and start treatment, thereby decreasing osteoporotic fracture rates.

This survey revealed that 13.6% of men and 8.5% of women aged ≥ 60 years had type 2 diabetes, and the percentage of these patients increased with age; in women, the percentage rose from 0.9% in the 40–44-year age group to 10.5% in the 80–84-year age group. In addition, 17.4% of hip fracture events developed among diabetic patients. Previous studies have shown that type 2 diabetes is associated with an increased risk of fracture despite high bone mineral density (BMD) [3]. In a meta-analysis of patients with type 2 diabetes, the risk of proximal femur fracture was found to be 1.38 (95% CI 1.25–1.53) [3], and the risk of distal radius, proximal humerus, heel, or vertebral body fractures was 1.2 (95% CI 1.01–1.5) [4]. According to a national diabetes database in Scotland, those with type 1 diabetes have a substantially elevated risk of hip fracture compared to the non-diabetic population. However, there was no elevation in risk among men, and the increase in risk in women was small, with an incidence risk ratio of 1.05 [5]. Our results showed that diabetic patients developed hip fractures more frequently than the population in Japan, with SIRs of 1.71 in men and 1.81 in women, and SIRs for radius and clinical vertebral fractures of around 1.2–1.3 in both men and women. We included patients who were diagnosed as having type 2 diabetes and were on medication, excluding diabetic patients treated with diet and exercise only. Therefore, the difference in fracture incidence can be seen more clearly in this study. An increased incidence of hip fractures can be seen in both men and women with type 2 diabetes, even among those in their late 40 s.

The prevalence of COPD is estimated to be 8.6% of the population aged ≥ 40 years, or approximately 5.3 million patients according to the Nippon COPD Epidemiology study in Japan [12]. However, the number of patients nationwide who were diagnosed as having COPD was 220,000 in 2017 according to a patient survey conducted by the MHLW [13], in which only 4% of the patients were treated. This study showed that approximately 460,000 people aged ≥ 50 years with the diagnosis of COPD were treated with LAMA or LABA, and the percentages of those who were diagnosed as having COPD and were receiving LAMA or LABA were 1.9% for men and 0.3% for women aged ≥ 60 years.

Several studies have shown a high fracture risk with COPD. A large case–control study (*n* = 124,655) in Denmark showed that the odds ratio (OR) of COPD and fracture was 1.19 [6]. A large multinational cohort study (GLOW) found that the hazard ratio of COPD for incident fracture was 1.2 [14]. Men with COPD or asthma had a 2.6- and 1.4-fold increased risk of vertebral and non-vertebral fractures, respectively, according to the Osteoporotic Fractures in Men [7]. A survey of Hong Kong Chinese people [15] indicated that patients with COPD developed non-vertebral fractures 1.74 times (1.14–2.68) more frequently than the community-dwelling Chinese. Our results showed that the SIRs of hip fracture in COPD patients were 1.35 in men and 1.6 in women. We also reported that male COPD patients developed significantly more clinical vertebral fractures, and the risks of radius and clinical vertebral fractures were higher in women with COPD than in the Japanese population.

This study showed that the percentage of GC users increased with age, reaching 2.5% among those in their late 80 s. It is well established that BMD decreases early after the start of GC use, and the risk of fractures increases sharply within 3 to 6 months of GC initiation [8]. The relationship between oral GC use and fracture risk has been revealed in the UK’s large General Practice Research Database [8]. Fracture risk was 1.3 times greater for overall fracture, 1.6 times for hip fracture, 1.1 times for wrist fracture, and 2.6 times for vertebral fracture. This study showed that the SIR for clinical vertebral fracture was the highest at 3.06 among male GC users, and it was 2.69 in women GC users among the three fracture sites.

A hip fracture incidence study was conducted using nationwide hospital- and clinic-based mail surveys in 2017 in Japan, which showed that the total number (95% confidence interval) of hip fractures was approximately 193,400 (187,300–199,500) [1]. We identified 169,200 hip fracture events, approximately 13% less than the results from the mail survey. Similarly, in 2012, hip fracture incidence was estimated using both the NDB [16] and a mail survey [17]. The number of hip fracture events was reported to be approximately 20% lower on using the NDB than that on using the nationwide mail survey at that time [16]. There are two possible explanations for this difference [16]. First, the recurrence of a hip fracture may have been missed on using the NDB because multiple fracture events in the same year were considered a single event in the NDB. Second, the response rate was 61.4% in the nationwide mail survey, wherein hip fracture cases might have been overestimated because incidences were estimated on the basis of the assumption that patients with hip fracture were equally likely to be seen at institutions that did or did not respond to the mail questionnaire survey.

The incidence of distal radius fractures has been reported using a complete enumeration survey for hospitals and clinics in a local city in Japan [18]. In that study, distal radius fractures accounted for approximately one-third of the overall radius of bone length. In this study, however, it is difficult to strictly classify distal radius fractures using ICD codes only, and we counted all potential distal radius fractures. Because we collected data on patients who had an ICD code of radius fracture and received medical procedure codes for invasive or non-invasive fixation of the forearm or surgery, we might have missed cases without such orthopedic procedures and surgery. The incidence of radius fractures was lower in this study than in previous reports and might have been underestimated.

The incidence of clinical vertebral fractures in this study was much lower than that reported previously in Japan [18–20]. Two [18, 20] of these previous studies were surveys from hospitals and clinics in local cities, and the other used medical care information from the National Health Insurance (NHI) or Senior Elderly Care System (SECS), which covers 86% of the city population [19]. In principle, most patients with vertebral fractures receive conservative treatments aimed at pain control and bone fusion with sedatives and trunk orthoses. It is difficult to distinguish patients with new or old vertebral fractures receiving sedatives using only the NDB. We identified patients with both ICD codes for vertebral fracture and trunk orthoses, such as Gibbs and splint treatment, as having a new vertebral fracture. We compared our results with the incidence rates from a previous study [18], in which clinical incident fractures were defined based on X-ray and physical examinations, as well as magnetic resonance imaging when conventional radiographs were insufficient for diagnosing a fresh fracture as a new fracture. The incidence rates in our study were about one-fifth lower, when compared with the previous study. Some reasons may explain the low incidence rates in this study. First, our study did not include new cases of clinical vertebral fractures that were treated conservatively, which includes bed rest, or sedatives only. Second, we counted cases with several vertebral fracture events in the same year as a single fracture event. This was because the MHLW Insurance Bureau considers patients with both vertebral fracture codes and orthoses codes during the same year as patients with a single fracture event during that year, even if they experienced several vertebral fracture events in the same year. Third, some diagnoses and orthoses codes may have been misclassified. However, we do not think that the eligibility criteria for new patients in this study were biased depending on whether patients were receiving medication for diabetes or COPD or were GC users. The incidence rates of clinical vertebral fracture and radius fracture were likely to be underestimated in this study. However, the SIRs for type 2 diabetes and COPD medication and GC users versus the Japanese population were not affected by the definition of fractures.

In this study, we found associations between patients medicated for diabetes or COPD and fracture risk. There have been several reported risk factors for fractures among patients medicated for diabetes or COPD. Steroid use, bronchodilator use, and smoking were risk factors found in patients with COPD [21–23], whereas falls, complications, and diabetes medications were risk factors found in patients with diabetes [24, 25]. However, from this study, we were unable to discern whether the associations were related to the medications itself or the underlying condition or risk factors for fracture.

The strength of this study was the use of data from NDB, which covers over 98% of the Japanese population. The medical insurance system in Japan is a “universal insurance system” in which individuals are obliged to take out some kind of public medical insurance and support each other’s medical expenses. This medical system allows people to access and to receive medical care and treatment using public insurance at any medical institution and at any time. Therefore, the result derived from the NDB will reflect the real world of medical treatment and conditions of the entire Japanese population. However, this study has some limitations. First, the data were from a single year without a washout period, as there was an exact date on the extracted data at first diagnosed. When using the claims database to check the incidence rate of fracture, it is ideal to set the fracture-free period (washout period) or clean period to establish new fracture cases with data from the previous year [26, 27]. Our data were based on the “first diagnosed” date; thus, patients who had a fracture in the previous year (i.e., end of December 2016) and consulted a clinic or hospital the next year (i.e., beginning of January 2017) were considered cases from 2017 although they developed a fracture in 2016. The second is the accuracy of the diagnosis of the NDB. In this study, to improve the accuracy of the diagnosis of disease using the ICD code, we used the billing codes for information on hospitalization, outpatient, surgery/medical treatment, drug name codes, in addition to disease ICD code. For example, we defined patients who were receiving diabetes medical treatment (oral and/or injection) with a diagnosis code of type 2 diabetes as diabetes on medication and patients who were receiving LAMA or LABA with the diagnosis code of COPD as COPD on medication. This study did not include patients with diabetes who were treated with diet and exercise treatment and those not using LAMA or LABA. Third, steroid users were defined as those who had been using oral steroids for 3 months or more using the 2016 and 2017 claims database. Fracture risk is associated with the amount of GC; however, we did not take into account the GC dose. Regardless, this study provides a real-world picture of fractures among patients who are prescribed drugs that are standardized for the treatment of diabetes or COPD and among GC users.

These findings from the national health receipt data from the National Database reveal that patients receiving medical treatment for diabetes or COPD and GC users have a higher risk of hip, distal radius, and clinical vertebral fractures compared to the Japanese population. The increased risk of hip fracture among diabetic patients can be seen even in those in their late 40 s.

## Supplementary Information

Below is the link to the electronic supplementary material.

Supplementary file1 (XLSX 49 KB)
